# A cryptic splice-altering *KCNQ1* variant in trans with R259L leading to Jervell and Lange-Nielsen syndrome

**DOI:** 10.1038/s41525-021-00183-y

**Published:** 2021-03-04

**Authors:** Mario Torrado, Germán Fernández, Christian A. Ganoza, Emilia Maneiro, Diego García, Natalia Sonicheva-Paterson, Isaac Rosa, Juan Pablo Ochoa, Luis Santomé, Elena Vasichkina, Lorenzo Monserrat

**Affiliations:** 1 Institute of Health Sciences, University of A Coruña, A Coruña, Spain; 2 Cardiovascular Genetics, Health in Code, A Coruña, Spain; 3 Almazov National Medical Research Centre, Saint Petersburg, Russia

**Keywords:** RNA splicing, Molecular medicine, Cardiovascular diseases, Clinical genetics

## Abstract

Here we report an infant with clinical findings suggestive of Jervell and Lange-Nielsen syndrome (JLNS), including a prolonged QT interval (LQTS) and chronic bilateral sensorineural deafness. NGS analysis revealed one known heterozygous pathogenic missense variant, KCNQ1 p.R259L, previously associated with LQTS but insufficient to explain the cardioauditory disorder. In a screening of proximal intronic regions, we found a heterozygous variant, *KCNQ1* c.1686−9 T > C, absent from controls and previously undescribed. Several splicing prediction tools returned low scores for this intronic variant. Driven by the proband’s phenotype rather than the neutral predictions, we have characterized this rare intronic variant. Family analysis has shown that the proband inherited the missense and the intronic variants from his mother and father, respectively. A minigene splicing assay revealed that the intronic variant induced an additional transcript, arising from skipping of exon 14, which was translated into a truncated protein in transfected cells. The splice-out of exon 14 creates a frameshift in exon 15 and a stop codon in exon 16, which is the last exon of *KCNQ1*. This mis-spliced transcript is expected to escape nonsense-mediated decay and predicted to encode a truncated loss-of-function protein, KCNQ1 p.L563Kfs*73. The analysis of endogenous *KCNQ1* expression in the blood of the proband’s parents detected the aberrant transcript only in the patient’s father. Taken together, these analyses confirmed the proband’s diagnosis of JLNS1 and indicated that c.1686−9 T > C is a cryptic splice-altering variant, expanding the known genetic spectrum of biallelic *KCNQ1* variant combinations leading to JLNS1.

## Introduction

Jervell and Lange-Nielsen syndrome (JLNS) is a recessive cardioauditory disorder characterized by long QT syndrome (LQTS) associated with severe bilateral sensorineural hearing loss. This rare condition, affecting 3–5 in one million children^1^, is caused by loss-of-function biallelic variants in the *KCNQ1* gene (90.5% of cases^2^), leading to JLNS type 1 (JLNS1, OMIM 220400), or in the *KCNE1* gene (9.5% of cases), leading to a similar syndrome known as JLNS2 (OMIM 612347). Accordingly, JLNS is suspected in a child with congenital deafness and a prolonged QT interval. The identification of biallelic, homozygous or compound heterozygous, pathogenic variants in either *KCNQ1* or *KCNE1* confirms the JLNS1 or JLNS2 diagnosis, respectively. Genetic diagnosis of JLNS is also relevant for appropriate patient management, taking into account that JLNS1 patients with *KCNQ1* variants are at higher risk, whereas *KCNE1* variants in JLNS2 patients are associated with a more benign course. In the largest JLNS study population^2^, it was found that JLNS1 patients had 6-fold greater risk of arrhythmic events than JLNS2 patients.

KCNQ1 and the regulatory subunit KCNE1 are voltage-gated potassium channel-interacting proteins required for the repolarization phase of the cardiac action potential. In the heart, KCNQ1 co-assembles with KCNE1 to form a channel complex that serves as the slow component of the delayed rectifier potassium current (I_Ks_), which is critical for the cardiac action potential^3,4^. Disturbances in I_Ks_ are clinically manifested by the presence of a long QT interval on a 12-lead electrocardiogram (ECG). While LQTS is associated with complex ventricular tachyarrhythmia, including ventricular tachycardia, torsade de pointes, and ventricular fibrillation or sudden death, the most common presentation of fetal LQTS is sinus bradycardia^5^. In the inner ear, the KCNQ1/KCNE1 potassium channel regulates endolymph recycling by controlling K^+^ secretion^6^; therefore, disruption of K^+^ recycling is responsible for the auditory phenotype in JLNS.

Most (74%^2^) biallelic *KCNQ1* variants leading to JLNS1 include at least one truncating variant which may interfere with or prevent channel assembly, while most of heterozygous nucleotide substitutions in *KCNQ1* leading to LQTS are missense variants with a dominant-negative effect, with incomplete penetrance^7^, since they are expected to co-assemble with normal subunits and interfere, to a variable extent, with channel function^2,8^.

In this work, we report an infant proband with clinical findings suggestive of JLNS who underwent genetic testing. Targeted next-generation sequencing revealed a single known pathogenic variant in the *KCNQ1* gene (p.R259L) in the heterozygous state. Detailed analysis of proximal intronic regions revealed the presence of an additional, previously undescribed variant in intron 13 (c.1686−9 T > C) of the *KCNQ1* gene, in compound heterozygosity with p.R259L. Although several in silico splicing tools predicted a neutral effect, and driven instead by the proband’s clinical phenotype, we have characterized this intronic variant by experimental pre-mRNA splicing assays, which have shown that the *KCNQ1* nucleotide substitution c.1686−9 T > C is a cryptic splicing-altering variant.

## Results

### Clinical characterization

**Fig. 1:**
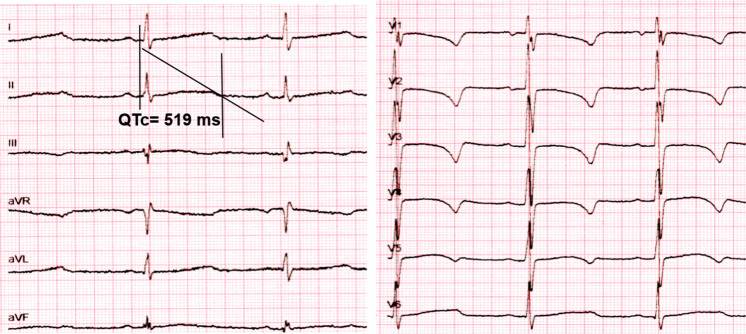
QTc value of the index patient. The QT interval corrected for heart rate (QTc) was measured in lead II from 12-lead electrocardiograms.

The proband was a 1-year-old male infant. His first clinical event was transient fetal bradycardia detected during routine fetal heart monitoring in week 40 of pregnancy. Cesarean delivery was performed due to suspected fetal distress. Physical examination and biometric parameters at birth were normal (weight: 3800 grams, height: 55 cm, Apgar score 7–8). During hospitalization, the patient did not experience new episodes of bradycardia and was discharged 4 days after birth. However, in the earliest months of life, paroxysmal events described as brief dissociative episodes were detected. The ECG showed a sinus rhythm of 86 beats per minute (considered bradycardia according to age), early-onset and broad-based T waves, with a prolonged QT interval. In this case, the QTc interval was 519 ms (Fig. 1). He had not received any QT-prolonging drugs and had no electrolyte abnormalities. Transthoracic echocardiography indicated a normal-size left ventricle, without structural abnormalities. A 24-h Holter recording was performed and showed a mean heart rate of 92 beats per minute. The maximum QT interval was 560 ms, and beat-to-beat instability in T-wave polarity and amplitude were observed. No complex ventricular arrhythmias were detected. Additionally, the patient underwent an audiological examination revealing chronic bilateral sensorineural deafness of fourth (IV) degree. Taken together, these clinical characteristics were suggestive of Jervell and Lange-Nielsen syndrome (JLNS).

No other family member suffered from hearing loss or has been diagnosed with a cardiac disorder, although the proband’s mother had a family history of early sudden death in her uncle and aunt. As the parents of an infant with JLNS may have long QT syndrome (LQTS), the proband’s first-degree relatives were evaluated. The mother’s ECG showed a QTc interval of 485 ms, and the T-wave morphology was suggestive of LQTS, but she was otherwise clinically asymptomatic. Both the proband’s father (QTc 458 ms) and sister (QTc 460 ms) had QTc at the upper limit of normal values and were also asymptomatic.

We next performed a genetic screening of the index patient to confirm the suspected JLNS diagnosis and identify the genetic origin of the disease.

### NGS analysis

Genomic DNA from the index patient was screened via targeted NGS analysis of genetic variants using a 218-gene panel including *KCNQ1* and *KCNE1*. After using a custom pipeline for filtering and classification of NGS data, relevant and suspected candidate variants were found in the *KCNQ1* gene, but not in *KCNE1* or in other genes in the panel. Out of a total of ten single-nucleotide variants identified by NGS in the *KCNQ1* gene, eight variants (namely #2, #3, #4, #5, #6, #7, #9, and #10 in Table 1) were discarded, since their allele frequency in the control population was higher than expected for disease or/and are classified as benign in the ClinVar database, leaving the variants #1 and #8 (see Table 1).

**Table 1:** Identified *KCNQ1* variants using targeted NGS.

#ID	GRCh38	GRCh37	Coding DNA	Protein	Location	Zygosity	Reads	SNP ID	AF gnomAD	ClinVar
	NC_000011.10	NC_000011.9	NM_000218.2	NP_000209.2						
#1	g.2572105 G > T	g.2593335 G > T	c.776 G > T	p.Arg259Leu	E5	HTZ	320	rs199472720	T = NA^a^	P
									A = 0.0000181	P/LP
#2	g.2768626 A > G	g.2789856 A > G	c.1515 − 218 A > G		i11	HZ	28	rs163161	G = 0.9952	B
#3	g.2768789 G > A	g.2790019 G > A	c.1515 − 55 G > A		i11	HTZ	255	rs2075870	A = 0.05429	NA
#4	g.2776007 G > A	g.2797237 G > A	c.1638G > A	p.(Ser546 = )	E13	HTZ	365	rs1057128	A = 0.2018	B
#5	g.2776075 G > A	g.2797305 G > A	c.1685 + 21 G > A		i13	HTZ	229	rs376557660	A = 0.0004125	NA
#6	g.2776090 A > G	g.2797320 A > G	c.1685 + 36 A > G		i13	HTZ	186	rs163150	G = 0.6682	NA
#7	g.2776200 G > C	g.2797430 G > C	c.1685 + 146 G > C		i13	HTZ	29	rs163149	C = 0.4947	NA
#8	g.2776977 T > C	g.2798207 T > C	c.1686 − 9 T > C		i13	HTZ	291	NA	NA	NA
#9	g.2777218 A > G	g.2798448 A > G	c.1732 + 186 A > G		i14	HZ	36	rs163148	G = 0.9979	B
#10	g.2847648 T > C	g.2868878 T > C	c.1795 − 119 T > C		i15	HTZ	92	rs3852520	C = 0.3997	B

#ID—Unique tag ID number assigned to each variant. The corresponding genomic (GRCh38 and GRCh37 assembly versions), coding DNA and protein NCBI Reference Sequences are indicated. Location—the exon (E) or intron (i) number are indicated.

*HZ* homozygous, *HTZ* heterozygous, *Reads* number of NGS reads covering the variant position, *AF* *gnomAD* population frequency in the gnomAD database, *ClinVar* ClinVar variant interpretation category, *P* Pathogenic, *P/LP* Pathogenic/Likely pathogenic, *B* Benign, *NA* not available.

^a^The population frequency for the G > T substitution is not available in gnomAD, the frequency of the G > A variant is shown. Although both variants share a common SNP ID (rs199472720), the G > A nucleotide change leads to a different amino acid substitution, p.Arg259His.

**Fig. 2:**
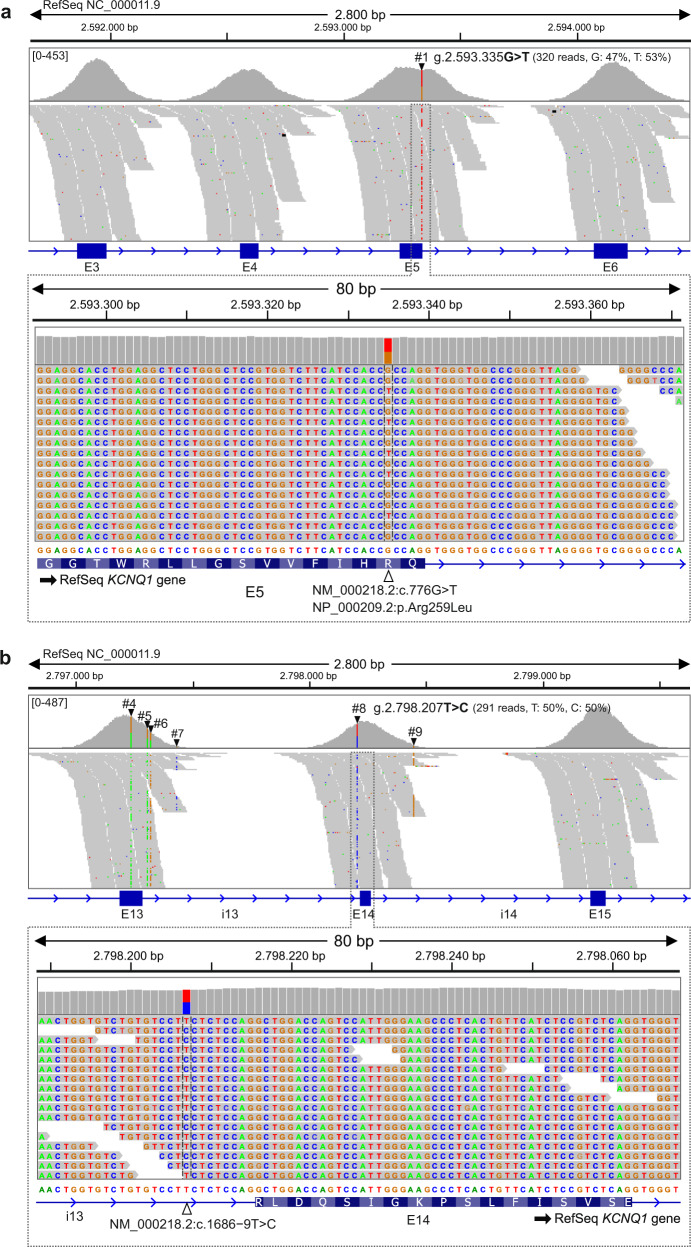
NGS data visualization and identification of *KCNQ1* variants. Graphics output of the IGV alignments of NGS reads from the index patient at two *KCNQ1* genomic positions leading to the identification of the pathogenic variant c.776 G > T in exon 5 (variant #1 in **a**) and a previously undescribed variant c.1686−9 T > C in intron 13 of the *KCNQ1* gene (variant #8 in **b**). A unique tag (#) ID number was assigned to each identified variant. The figure was assembled using scalable vector graphic files exported from the IGV application. NGS reads (horizontal grey bars) alignments to the reference genome sequence (RefSeq NC_000011.9) are displayed at two scales, 2800 bp and a zoomed version at 80 bp. Both nucleotide variants #1 and #8 were detected in the heterozygous state and the percentage of reads corresponding to each alternative nucleotide are shown with round brackets. Coverage graphs are shown with grey vertical bars and the coverage range is indicated in square brackets. Exon (E)–intron (i) structure of each displayed *KCNQ1* regions are represented with blue boxes and lines, respectively. The complete descriptions of the ten *KCNQ1* variants identified in the index patient’s genomic DNA by targeted NGS are shown in Table 1 with the same tag (#) ID numbers used in this figure.

The exonic variant #1 (c.776 G > T), located in exon 5, is a missense variant which leads to an arginine-to-leucine amino acid substitution at position 259 (p.Arg259Leu or p.R259L) of the KCNQ1 protein. In this variant, the reference nucleotide G at position c.776 changes to T in one allele of the patient (Fig. 2a). The KCNQ1 p.R259L variant has been reported numerous times in association with LQTS and is classified as pathogenic in the ClinVar database (variation ID: 53102), and as such it would explain the LQTS of the index patient, but not the JLNS.

As shown in the Table 1, it is worth noting that the variant #8 (c.1686−9 T > C) is the only one that has not been described or reported in the literature or databases, including the Single Nucleotide Polymorphism database (dbSNP) and the Genome Aggregation Database (gnomAD). *KCNQ1* c.1686−9 T > C is an intronic variant detected in the heterozygous state and located in intron 13, nine nucleotides upstream of the splice acceptor site of exon 14 (Fig. 2b).

### Splicing predictions

We have used several splicing tools in an attempt to predict the consequences of the intronic variant. The Alamut in silico splicing analysis of the c.1686−9 T > C variant returned low scores in all four prediction methods, although all of them pointed to a slightly reduced efficiency (ranging from −4.9% to −1.7%, Supplementary Fig. 1) of the natural splice acceptor site at c.1686. In the light of these predictions, it is relevant to note that the c.1686−9 T > C variant is located within a region of pyrimidine (T, C in DNA) abundance, the polypyrimidine tract, which precedes the essential AG dinucleotide at the acceptor site. The in silico prediction of the splicing consequences of a T > C nucleotide change at position −9 represents a challenge for position-specific weight matrix algorithms, taking into account, first, that both T and C are similarly represented (44% and 38%, respectively) at position −9 in human splice acceptor sites^9^ and, second, that the T > C variation at *KCNQ1* c.1686−9 does not create a new recognizable splice site. Accordingly, the deep learning-based tool to identify splice variants SpliceAI also returned only a very low score for the delta score acceptor loss (Supplementary Fig. 2). Contrary to these in silico predictions and driven by the patient’s phenotype instead, we have considered that the rare *KCNQ1* c.1686−9 T > C variant could be a suitable candidate if two criteria are met: first, be present in compound heterozygosity with p.R259L, and second, be able to affect RNA splicing in experimental assays. Therefore, we performed additional genetic and functional analyses. Interestingly, using a different in silico approach, SpliceAid analysis of RNA target motifs bound by splicing proteins, predicted that the variant *KCNQ1* c.1686 − 9 T > C abolishes a binding site motif for the polypyrimidine tract binding protein 1, which is present only in the wild-type sequence (Supplementary Fig. 3).

### Family genetic testing

**Fig. 3:**
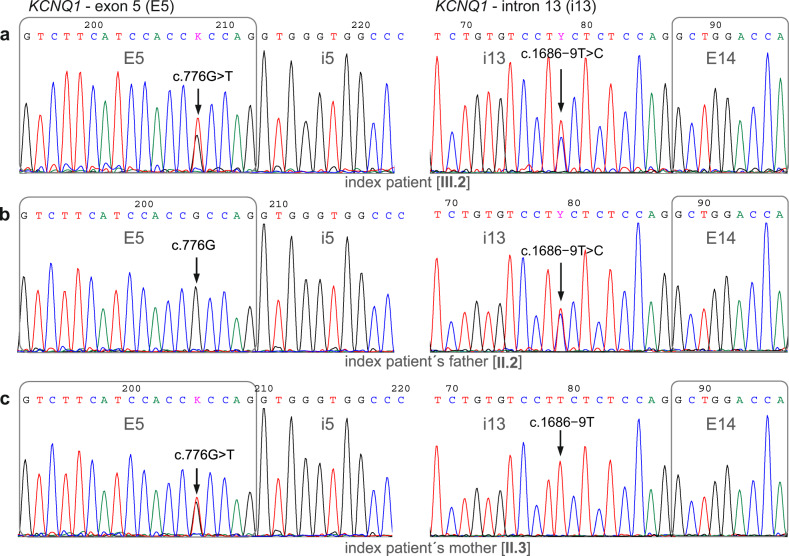
Parental origin of the pathogenic c.776 G > T and the candidate c.1686−9 T > C *KCNQ1* variants identified in the index patient. DNA sequencing chromatograms of the *KCNQ1* gene exon 5 (left) and intron 13 (right) from the index patient (**a**), father (**b**), and mother (**c**). The index patient’s variant c.776 G > T in exon 5 was detected in his mother, whereas the c.1686−9 T > C variant in intron 13 was detected in his father.

**Fig. 4:**
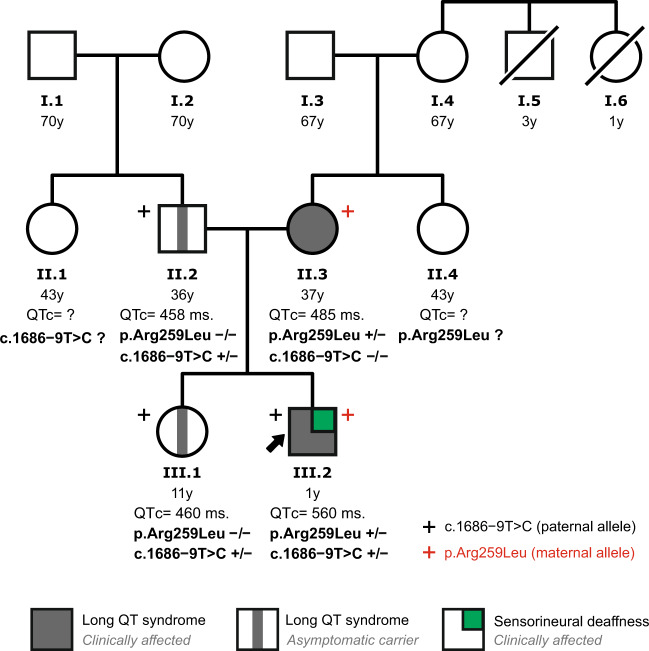
Family pedigree of the index patient. Available *KCNQ1* genotype and clinical data are shown. Arrow indicates the index patient.

We first determined the parental origin of both the exonic c.776 G > T (#1, p.Arg259Leu) and the intronic c.1686−9 T > C (#8) *KCNQ1* variants identified in the index patient (Fig. 3). The Sanger DNA sequencing analysis of the family has shown that the index patient (III.2) inherited the exonic c.776 G > T variant from his mother (II.3), while the intronic c.1686−9 T > C variant was inherited from his father (II.2). Thus, the family screening showed that the proband is compound heterozygous for the variants #1 and #8 in the *KCNQ1* gene, harboring the two variants located on different alleles. The family pedigree, summarizing the genotype and clinical data, is shown in Fig. 4. The proband’s mother (QTc 485 ms) was a carrier of the missense variant p.Arg259Leu, while the proband’s father and sister (both with a borderline QTc interval) were carriers of the intronic variant.

### In vitro assay of pre-mRNA splicing

**Fig. 5:**
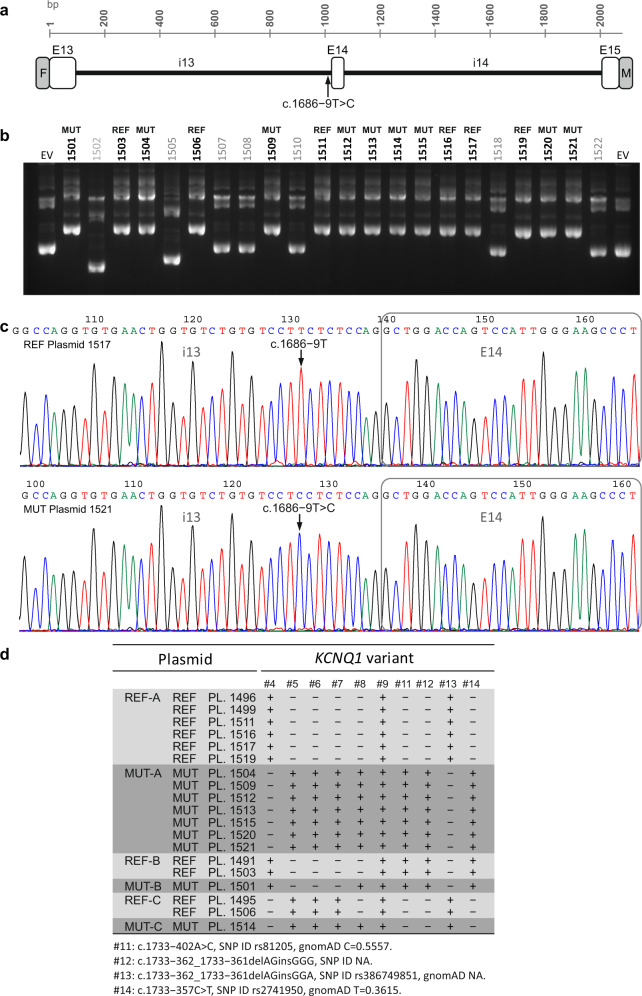
Generation and characterization of patient-specific *KCNQ1* minigene constructs. **a** Structure of the 2078-bp *KCNQ1* minigene fragment from exon 13 (E13) to 15 (E15), which includes the c.1686−9 T > C variant in intron 13, that was amplified from the patient genomic DNA and cloned in-frame to both the FLAG (F) and Myc (M) epitope tags of the expression vector. **b** Identification of positive minigene constructs by agarose electrophoresis of plasmid DNA isolated from 22 ampicillin-resistant bacterial clones. Plasmids with bold numbers, displaying reduced electrophoretic mobility as compared to the empty vector (EV), were identified as positives (containing the insert) and sequenced. Plasmids with the reference T or alternative C nucleotide, corresponding to the variant c.1686−9 T > C, were classified as REF or MUT, respectively, as indicated on the top of each lane. **c** DNA sequencing chromatogram of REF 1517 and MUT 1521 plasmids. The arrows mark the position of the affected nucleotide. **d** *KCNQ1* variants identified by Sanger sequencing of 19 positive plasmids. For each variant, the presence of the reference (−) or the alternative (+) nucleotide in each plasmid sequence is indicated. Variants are labeled with the same tag (#) ID numbers used in Table 1 and Fig. 2.

Then, we performed a functional analysis of the intronic c.1686−9 T > C variant by an in vitro assay of pre-mRNA splicing by minigene expression. As shown in Fig. 5, patient-specific minigene expression plasmids carrying a *KCNQ1* gene fragment (Fig. 5a) were constructed, characterized by gel electrophoresis (Fig. 5b, Supplementary Fig. 4) and sequenced (Fig. 5c), in order to identify the resulting positive clones carrying the normal T (labeled as REF) or the alternative C (labeled as MUT) nucleotide at position c.1686−9. Finally, we have obtained 19 positive plasmids, which were subjected to full insert sequencing (Fig. 5d). As expected, not only the candidate variant #8 (c.1686−9 T > C) but also variants from #4 to #9, which had been previously identified in the patient’s genomic DNA by NGS, were present in the plasmids. Full DNA sequencing of plasmid inserts revealed four additional heterozygous *KCNQ1* variants (labeled as #11, #12, #13, and #14 in Fig. 5d) located in the middle of intron 14 and out of coverage of the NGS probes. Most plasmids (Fig. 5d), categorized as REF-A (6 plasmids) or MUT-A (7 plasmids), were identical at the nucleotide level within each group, suggesting that they represent each patient’s allelic genotype. The other plasmids, categorized as REF-B/MUT-B and REF-C/MUT-C, displayed alternative configuration of *KCNQ1* variants, probably generated by PCR-mediated recombination, and are identical within the B or C group, with the only exception of variant #8. A pair of REF-MUT plasmids from each A, B, and C categories were selected for expression studies, so that the functional consequences of the candidate variant #8 (c.1686−9 T > C) on RNA splicing could be analyzed in the presence or absence of other proximal and naturally occurring variants that were incorporated into the sequence of the minigene plasmids from the patient genomic DNA.

**Fig. 6:**
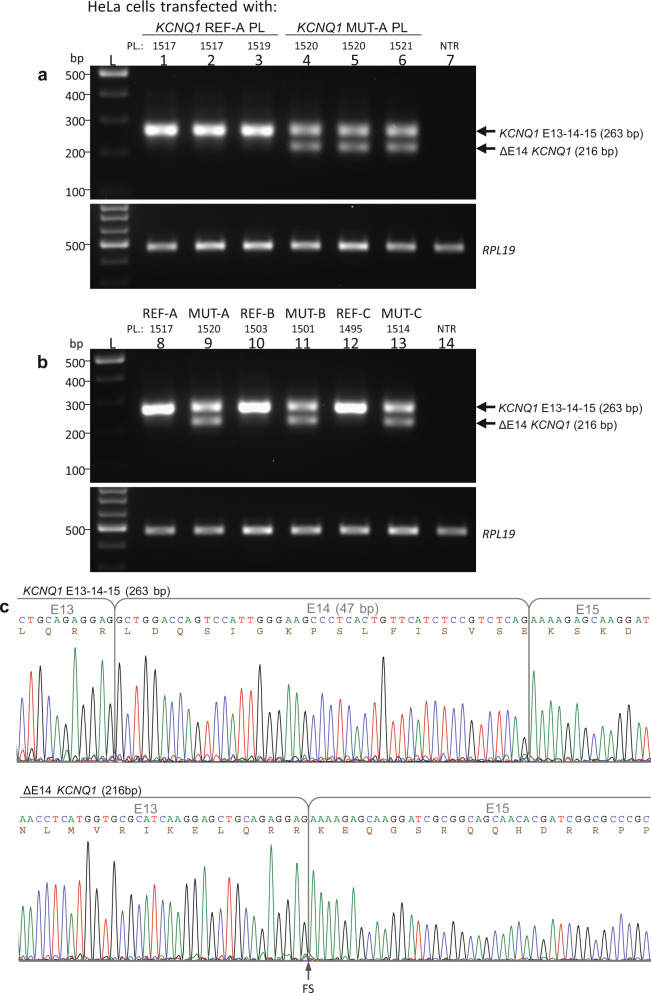
RT-PCR analysis of HeLa cells transfected with *KCNQ1* c.1686−9 T > C minigene plasmids. The agarose gel electrophoresis of minigene-derived *KCNQ1* and the endogenous *RPL19* RT-PCR products from two transfection experiments are shown in **a** and **b** using the indicated reference (REF) or mutant (MUT) *KCNQ1* minigene plasmids (as described in Fig. 5). Amplification of *KCNQ1* cDNA was performed with a forward primer located in E13 and a reverse primer in the vector-derived Myc epitope. Each RT-PCR lane (1–14) corresponds to a cell culture well processed independently. The amplification of *RPL19* was used as a cDNA normalization control. NTR nontransfected cells, L DNA ladder. **c** DNA sequencing chromatograms of the longer 263-bp *KCNQ1* band corresponding to the normal mRNA including exons 13–14–15 and the shorter 216-bp band with the complete skipping of exon 14 (ΔE14 *KCNQ1*). The deduced amino acid sequence is shown below the nucleotide sequence, as displayed by the Chromas software. The skipping of E14 introduces a frameshift (FS) at the start of E15. Full-size RT-PCR gel images are presented in Supplementary Fig. 5.

As shown by the RT-PCR analysis of the RNA isolated from cells transfected with the different *KCNQ1* REF plasmids (from A–B–C categories), all of them yielded a single band of the expected size (263 bp, Fig. 6a, b, Supplementary Fig. 5) corresponding to the normal inclusion of exons 13–14–15 and removal of the intervening introns 13 and 14. The Sanger sequencing of these RT-PCR products confirmed that the exons were correctly ligated together (Fig. 6c, top). In contrast, cells transfected with *KCNQ1* MUT minigenes expressed, in addition to the normal RNA, a shorter mis-spliced transcript (216 bp, skipping of exon 14, ΔE14, Fig. 6a, b). DNA sequencing confirmed the exclusion of 47 bp corresponding to the complete E14 in the 216 bp RT-PCR band (Fig. 6c, bottom). Cells transfected with the MUT plasmids expressed lower levels of the normal transcript (about 50%, Student’s *t*-test *p* < 0.01) as compared to the equivalent bands transcribed from the REF plasmids, as estimated by densitometry of the amplified bands. The two RNA forms derived from the MUT plasmids have been detected in a ratio of 68% (normal transcript, 263 bp) and 32% (ΔE14, 216 bp). In summary, the splicing assay by the expression of patient-specific *KCNQ1* minigenes indicated that the *KCNQ1* c.1686−9 T > C variant #8, but none of the other nine variants (#4, #5, #6, #7, #9, #11, #12, #13, and #14, see Fig. 5d) present in the plasmids, induces a mis-spliced transcript by skipping of E14, promoting the production of a mixture of normal and exon-skipped transcripts.

Full-length sequence analysis of the ΔE14 transcript has shown that the skipping of E14 creates a frameshift at the beginning of E15 and a premature termination codon (PTC) in E16, 79 nucleotides before reaching the natural termination codon in the same last E16. The aberrant protein would lose the last 114 amino acids of the C-terminal domain (from Leu563 to Ser676), which would be replaced by 72 new amino acids (p.L563Kfs*73, Supplementary Fig. 6). Since the PTC is located in the last E16, it is not expected that the mis-spliced transcript would be a target or activate the nonsense-mediated mRNA decay (NMD).

**Fig. 7:**
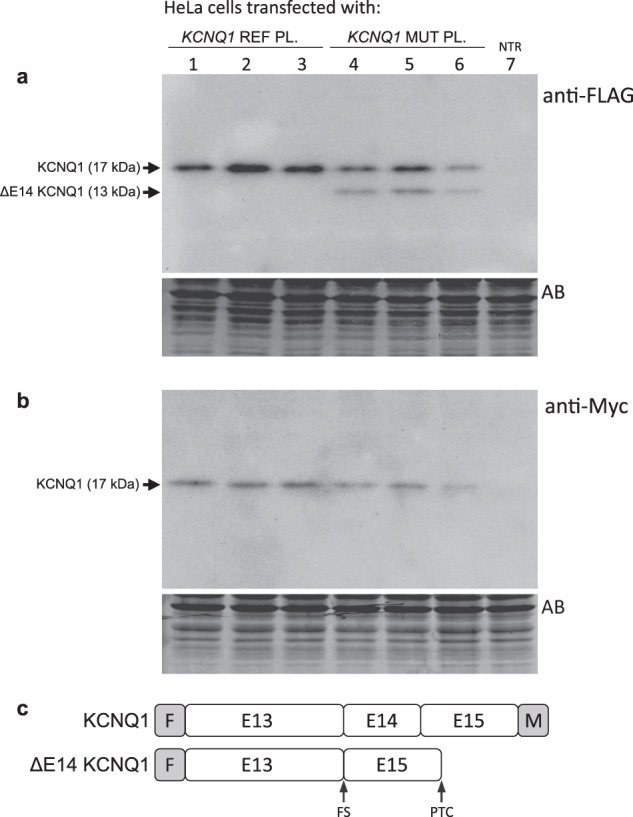
Western blot analysis of HeLa cells transfected with *KCNQ1* c.1686−9 T > C minigene plasmids. *KCNQ1* Reference (REF) or Mutant (MUT) minigene plasmids (PL) were transfected into HeLa cells (in triplicated wells) as indicated. Cell lysates, derived from the same experiment and processed in parallel, were analyzed by western blotting with mouse monoclonal anti-FLAG (**a**) or anti-Myc (**b**) antibodies 48 h after transfection. NTR nontransfected cells, AB membrane stained with Amido Black 10B after immunostaining. **c** Structure of minigene-derived KCNQ1 and ΔE14 KCNQ1 proteins as predicted by sequence analysis of the corresponding cDNAs. F FLAG epitope, M Myc epitope, FS Frameshift, PTC premature termination codon.

We next analyzed if the mis-spliced ΔE14 *KCNQ1* transcript could be translated into protein products in transfected cells. Biological replicates of HeLa cells were transfected with the same *KCNQ1* plasmids used in the experiment of Fig. 6a and analyzed by SDS-PAGE and western blotting (WB) as shown in Fig. 7 (and Supplementary Fig. 7). The anti-FLAG antibody (Fig. 7a) recognized the FLAG-KCNQ1 fusion proteins encoded by both the reference and mutant plasmids, as a band with an apparent molecular weight (MW) of 17 kDa, translated from the normal transcript. An additional, shorter protein of about 13 kDa was detected in cells transfected with the mutant plasmids, but not in cells transfected with the reference plasmids. The difference in the apparent MW between the normal and the shorter proteins matched the expected difference as a result of the skipping of E14. As estimated by densitometry of the WB bands, cells transfected with the mutant plasmids displayed reduced levels (about 44%, Student’s *t*-test *p* < 0.01) of the normal protein as compared to the equivalent bands derived from the REF plasmids. In addition, the two protein products derived from the MUT plasmids have been detected in a ratio of 66% (17-kDa protein) and 34% (13-kDa protein). Thus, the relative protein levels determined by WB were in good agreement with the mRNA expression data previously obtained by semiquantitative RT-PCR.

All stable proteins (normal or aberrant) encoded by the minigenes are expected to be fused to the N-terminal FLAG epitope. However, only those proteins that are translated from mRNAs with the normal reading frame are expected to be fused to the C-terminal Myc epitope. We have used this dual-tagged expression of N-terminal FLAG and C-terminal Myc system to further verify the identity and origin of the two FLAG-positive proteins. It should be noted that the skipping of exon 14 in the transcript encoded by the mutant minigene (which included exons 13–14–15, see Fig. 5a) is predicted to introduce a PTC after the out-of-frame translation of E15 and before reaching the Myc epitope. As shown in Fig. 7b, the anti-Myc-tag antibody recognized the normal larger protein, but not the FLAG-positive shorter one, present in the cells transfected with the MUT minigenes. This experiment has provided further evidence that the REF minigene produces a single protein, positive for both FLAG and Myc epitopes, which are translated from the normal transcript, whereas the MUT minigene produces an additional, shorter protein, positive for FLAG but negative for Myc tag, which are translated from the ΔE14 transcript (Fig. 7c).

Taken together, these in vitro splicing assay results indicated that, in transfected cells, the variant *KCNQ1* c.1686−9 T > C induces an alternative splice-in or splice-out of E14, transforming the constitutive E14 into an alternative exon and resulting in a mixture of normal (at reduced levels) and out-of-frame exon-skipped transcripts, which escape NMD and are effectively translated into protein.

### Endogenous *KCNQ1* expression

**Fig. 8:**
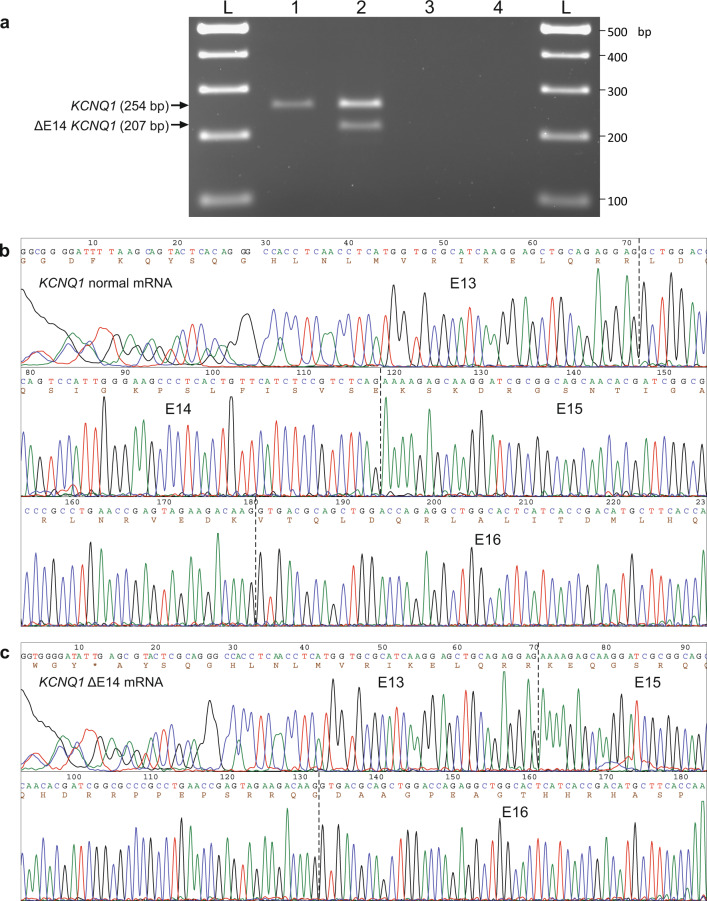
RT-PCR analysis of endogenous expression of *KCNQ1* in the blood of the index patient’s parents. **a** Agarose gel electrophoresis of RT-PCR products of blood RNA isolated from the index patient’s mother (lane 1) and father (lane 2). Amplification of *KCNQ1* cDNA was performed with a forward primer located in E13 and a reverse primer in E16. Negative controls were loaded in lane 3 (-RT) and 4 (nontemplate). The upper bands (254 bp) correspond to normal *KCNQ1* mRNA and the lower one (207 bp) to the ΔE14 *KCNQ1* aberrant mRNA. Below are shown the Sanger sequencing chromatograms of the 254 bp (**b**) and 207 bp (**c**) RT-PCR bands from the index patient’s father. The exon-exon junctions are marked with vertical dotted lines, and the number of each exon is indicated (E13-E16). The deduced amino acid sequence is shown below the nucleotide sequence, revealing the frameshift at the start of E15 that was created by the skipping of E14. Full-size RT-PCR image is shown in Supplementary Fig. 8.

Finally, with the aim of obtaining evidence that our in vitro assay of pre-mRNA splicing by the expression of minigenes could reflect the molecular consequences of *KCNQ1* c.1686−9 T > C in the patient, we performed an additional RT-PCR analysis of endogenous *KCNQ1* expression. For this analysis, taking into account the young age of the patient, the blood of the proband’s parents was used as the tissue source, since we have previously shown that the father, but not the mother, is a carrier of the c.1686−9 T > C variant. As shown in Fig. 8, in addition to the expected normal *KCNQ1* RT-PCR bands (254 bp), an additional, shorter band (207 bp) was detected in the blood of the proband’s father, but not in the mother. Sanger DNA sequencing revealed the splice-out of 47 bp corresponding to the complete E14 in the 207 bp mis-spliced RT-PCR band. These results confirmed that, in different cell hosts (in vitro and in vivo), the *KCNQ1* nucleotide substitution c.1686−9 T > C acts as a cryptic splicing-altering variant promoting skipping of exon 14.

## Discussion

In this work, we present the characterization of an intronic variant in *KCNQ1*, c.1686−9 T > C, that was detected on opposite chromosomes with the known pathogenic variant KCNQ1 p.Arg259Leu (p.R259L), in an infant patient with clinical findings of JNLS, including a prolonged QT interval and chronic bilateral sensorineural deafness. The proband underwent NGS genetic testing to confirm the diagnosis of JLNS and identify the genetic origin the disease. We have identified the *KCNQ1* c.1686−9 T > C variant only in the family described here, with several members affected. The intronic variant is absent from controls and although its frequency is unknown, it is expected to be extremely rare since it has neither been reported in the literature nor in the Genome Aggregation Database (gnomAD).

The heterozygous variant KCNQ1 p.R259L^8,10,11^ and also other substitutions affecting the same amino acid, p.R259C^8,12,13^ and p.R259G^14^, have been classified as pathogenic, leading to LQTS^15^. In addition, the pathogenic p.R259H variant has been associated with short QT syndrome^16,17^. These studies have revealed the essential role of the arginine residue at position 259 to maintain KCNQ1 functions. Further experimental studies have shown that R259 is part of an interaction domain that binds the membrane lipid PIP2, a factor required for channel function^18,19^. The variant p.R259G was also identified in a JLNS1 patient in association with a frameshift variant^14^. To our knowledge, the p.R259L variant has not been previously described in a JLNS1 patient, but it was reported in compound heterozygosity with another pathogenic missense variant, p.V524G, in an LQTS patient, with the auditory phenotype intact^20^. Moreover, in that retrospective study^20^, from a total of 15 patients that harbored rare putative pathogenic variants on both *KCNQ1* alleles, 11 presented without the sensorineural deafness associated with JLNS1. These findings highlighted that biallelic pathogenic *KCNQ1* variant combinations leading to sensorineural deafness may have additional requirements, which not all pathogenic combinations meet, with a marked preference (79%) toward truncating (nonsense, frameshift, or splicing) versus nontruncating (missense or in-frame deletion)^20^.

Taking into account that the intronic variant was the only candidate found, in compound heterozygosity with p.R259L, we performed RNA splicing assays to address its functional implications. The in vitro assay of pre-mRNA splicing by *KCNQ1* minigene expression has shown that the c.1686−9 T > C variant weakened the natural splicing at the acceptor site of exon 14 (E14) with two major effects, reducing normal transcript levels as compared to the reference wild-type control while also promoting the production of an additional, shorter mis-spliced transcript by skipping of exon 14 (ΔE14). In addition, in cells transfected with the mutant minigene, the ratio of normal/truncated proteins detected by western blot was similar to the ratio normal/ΔE14 transcripts detected by RT-PCR, indicating that both transcripts are translated into proteins with similar efficiencies. In fact, it is not predicted that the ΔE14 transcript, derived from the mutant minigene, undergoes NMD, since out-of-frame skipping of E14 creates a premature termination codon (PTC) located in the last minigene coding exon, after the out-of-frame translation of E15 and before reaching the engineered minigene stop codon placed after the C-terminal Myc-epitope tag.

Similarly, in the full-length *KCNQ1* gene, the splice-out of E14 creates a PTC in the last exon, E16, after the out-of-frame translation of E15 and before reaching the *KCNQ1* natural stop codon, which is also located in E16. Thus, it is not predicted that the ΔE14 transcript carrying a PTC in the last coding exon undergoes NMD. The presence of the endogenous ΔE14 transcript in the blood of the proband’s father (carrier of the *KCNQ1* c.1686−9 T > C variant), on one hand, confirmed the in vitro results and, on the other hand, supported the prediction that the mis-spliced transcript may at least partly escape NMD in vivo. Thus, it is expected that the ΔE14 transcript with a PTC within the last coding E16 would lead to a C-terminal truncated protein that retains the six transmembrane domains that constitute the voltage-sensor and pore domains, p.L563Kfs*73. The lost protein region (from L563 to S676) includes the KCNQ1 fourth helix D (residues 587–620) of the C-terminal assembly domain, which forms a coiled-coil structure essential for channel tetramerization^21^. Numerous pathogenic variants leading to LQTS and/or JLNS, including 20 missense and 10 frameshift variations^15^, have been identified downstream of L563.

Importantly, it has been reported that even small amounts of the normal-spliced *KCNQ1* transcript remaining in a homozygous c.387−5 T > A patient are able to preserve normal hearing function, although not cardiac function, thus leading to LQTS but not JLNS^22^. The c.387−5 T > A variant induces predominantly skipping of E2 (ΔE2), while maintaining about 10% of the normal-spliced transcript. The aberrant *KCNQ1* ΔE2 transcript introduces a PTC in E5, after the predicted out-of-frame translation of E3 and E4. Therefore, it is expected that the ΔE2 aberrant *KCNQ1* mRNA will be a target of NMD, promoting its degradation in vivo, preventing the synthesis of aberrant truncated proteins, and leaving a reduced amount of normal full-length KCNQ1 protein, which was sufficient to maintain normal hearing function. Furthermore, the reported functional analysis^22^ has shown that if the ΔE2 aberrant transcript had escaped NMD, it would produce a non-functional truncated protein without a dominant-negative effect. In our case, although the alternative mis-splicing of E14, promoted by the c.1686−9 T > C variant, is expected to maintain a relatively large amount of the normal transcript, it was intriguingly unable to preserve normal hearing in our patient. We considered that the essential difference between the ΔE2 and the ΔE14 aberrant transcripts (both generated by exon skipping) is the relative position of the PTC, so it is expected that the ΔE2 transcript, but not ΔE14, will be a target of NMD. Accordingly, although not yet proven in vivo, we suggested that the translation of the ΔE14 *KCNQ1* transcript, derived from the paternal allele in our index patient, would lead to the production of a loss-of-function C-terminal truncated KCNQ1 protein (p.L563Kfs*73), which might have prevented that the normal hearing requirements could be maintained by the remaining normal KCNQ1 protein. The truncated protein is expected to retain the N-terminal and the six transmembrane domains but, lacking the C-terminal tetramer assembly domain, would be unable to contribute to the assembly with the remaining normal protein from the paternal allele or the p.R259L mutant protein from the maternal allele, preventing KCNQ1 tetramerization and interfering with channel formation and function.

We consider that the highly specific cardioauditory phenotype of the index patient represents important evidence in support of a dominant-negative effect against the remaining functional protein coming from the *KCNQ1* c.1686−9 T > C paternal allele. Otherwise, in the absence of a dominant-negative effect, it is expected that even a small amount of functional protein would be sufficient to preserve normal hearing, which is not the case in our index patient. Additional functional experiments using cell or animal models may provide further insight into the molecular mechanism of this cryptic variant mediated by the mutant protein KCNQ1 p.L563Kfs*73, in the presence or absence of KCNQ1 p.R259L.

Our work has shown that the consequences of the rare intronic variant *KCNQ1* c.1686−9 T > C, which neither abolished the canonical splice sites nor created a new recognizable splice acceptor/donor site, are particularly difficult to predict using *in silico* analyses. We have used several computational prediction tools, and all of them returned low scores below the significance threshold. It is worth noting that SpliceAI has been successfully used to identify cryptic splice sites when intronic variants in the *MYBPC3* gene created new essential AG dinucleotide acceptor sites, even if they are located distant (at −80, −52, or −36) from the natural splice acceptor sites^23^, as previously shown with RNA assays^24–26^. In our case, the output obtained by a different approach, SpliceAid, pointed out that the mechanism that could explain the mis-splicing induced by *KCNQ1* c.1686−9 T > C is related to the alteration of the binding site motifs of regulatory splicing factors in the polypyrimidine tract of the splice acceptor site of intron 13.

In this study, driven by the index’s patient phenotype, it was mandatory to perform functional analysis to address the consequences of the *KCNQ1* c.1686−9 T > C variant. Our in vitro splicing assay of patient-specific minigene expression, with a structure of three-exon and two-intron, has identified precisely the aberrant ΔE14 RNA, which was further confirmed by endogenous expression analysis. Moreover, with the use of this system, we were able to specifically identify that the c.1686−9 T > C variant was the cause of the observed splicing defect, but not the other nine variants present in the plasmids and that were all incorporated into the constructions from the patient’s genomic DNA. In addition, the dual-tagged minigene expression allowed us to distinguish protein products that were translated from transcripts with preserved or altered reading frames.

Since we did not provide evidence of co-segregation in different families, and following the American College of Medical Genetics guidelines^27^, the *KCNQ1* c.1686−9 T > C variant was classified as likely pathogenic based on the following criteria: PS3 (functional studies supportive of a damaging effect on the gene or gene product), PM2 (the variant is absent from controls in gnomAD) and PM3 (for recessive disorders, detected on opposite chromosomes with a pathogenic variant, which requires testing of parents to determine phase).

In summary, the genetic and molecular analyses of this work have confirmed the index patient’s diagnosis of Jervell and Lange-Nielsen syndrome 1 (JLNS1) by the identification of biallelic variants in the *KCNQ1* gene, p.R259L and a previously undescribed intronic variant c.1686−9 T > C. Our molecular study indicated that the intronic *KCNQ1* c.1686−9 T > C variant is a cryptic splice-altering variant that promotes the production of out-of-frame exon-skipped transcripts, which are expected to escape NMD and predicted to encode a truncated loss-of-function protein. These results expand the known genetic spectrum of biallelic pathogenic *KCNQ1* variant combinations that, in addition to cardiac disorders, lead to sensorineural deafness in JLNS1 patients, and they also highlight the relevance of functional studies of intronic variants driven by the patient’s phenotype.

## Methods

### Patient consent for research and publication

Written informed consent was obtained from the minor’s parents and all family participants for the genetic analyses and for the publication of anonymized data obtained through the clinical characterization and the scientific research carried out. Ethical approval (n° 1906-20) has been obtained from the Ethics Committee of the Almazov National Medical Research Centre. The study was conducted in accordance with the Declaration of Helsinki.

### Clinical characterization

The one-year-old male proband has been clinically evaluated during the first months of life since the detection of signs of fetal distress during routine fetal heart monitoring in the late gestational stage. Transthoracic echocardiography, ECG and continuous Holter recording were performed. The patient underwent an audiological examination including: brainstem auditory evoked potentials, auditory steady-state response, transient-evoked otoacoustic emission and impedancemetry. The QT interval corrected for heart rate (QTc) was measured in lead II from 12-lead electrocardiograms with the use of Bazett’s formula^28^. The finding of a cardioauditory disorder with frequent genetic basis led to the genetic and clinical investigation of his first-degree family members. Major clinically relevant events in the family history were collected and annotated in the family pedigree.

### Genomic DNA purification

The patient’s and the relatives’ genomic DNA was purified from peripheral blood on the QIAsymphony SP robot using the QIAsymphony DNA midi Kit (Qiagen). Eluted genomic DNA was quantified using Nanodrop 1000 Spectrophotometer (Thermo Fisher Scientific), and DNA integrity was determined on the TapeStation 2200 automated electrophoresis unit (Agilent Technologies) following the manufacturer’s instructions.

### Targeted next-generation sequencing

A custom 218-gene arrhythmia general panel including *KCNQ1* and *KCNE1* (see Genetic Testing Registry at NCBI, www.ncbi.nlm.nih.gov/gtr/tests/530668/) was used for targeted Next Generation Sequencing (NGS) of all exons and intronic boundaries of the index patient’s genomic DNA. The capture of genomic regions of interest was performed using Sure-Select XT Target Enrichment Kit for Illumina paired-end multiplexed sequencing method (Agilent Technologies) and sequenced using the HiSeq 1500 platform (Illumina) following Illumina protocols. Bioinformatics analysis was performed by means of a custom pipeline including software for variant calling, genotyping, and annotation. Filtering of variants was performed using in-house datasets, the database of single-nucleotide polymorphisms (www.ncbi.nlm.nih.gov/SNP), the genome aggregation database (https://gnomad.broadinstitute.org), and the Health in Code database. Visualization of NGS data aligned to the human reference genome (GRCh37/hg19) was performed using the Integrative Genomics Viewer (IGV^29^), and nucleotide variants were named according to both GRCh37/hg19 and GRCh38/hg38 human genome assembly versions.

### Family genetic testing

Sanger DNA sequencing was used for independent confirmation of NGS variants, family testing and phase determination of two *KCNQ1* variants identified in the index patient, located in exon 5 (c.776 G > T) and intron 13 (c.1686−9 T > C). *KCNQ1* exon 5 was amplified from genomic DNA using forward primer EX5-FW (GAC ATA TAC CCA GCC TCC CCA) and reverse primer EX5-RV (GCG CAT CTC AAG CTG TCC TAG T), located in upstream and downstream introns, respectively. *KCNQ1* exon 14 along with its proximal intronic regions was amplified using forward primer EX14-FW (GTC AAG CTG TCT GTC CCA CAG A) and reverse primer EX14-RV (TTT CAT GTC ATG CAC TTT GGA G), located in intron 13 and 14, respectively. All primers are indicated from 5’ to 3’. PCR was performed with KAPA2G Fast HotStart ReadyMix (Roche), and the resulting PCR reaction was subjected to an enzymatic clean-up prior to sequencing using exonuclease I and thermosensitive alkaline phosphatase (Thermo Scientific) following manufacturer’s protocols. Forward and reverse sequencing reactions were performed using Big Dye Terminator v3.1 Cycle Sequencing kit (Applied Biosystems) and the same primers used in the PCR. The cycle sequencing products were purified with BigDye XTerminator Purification kit (Applied Biosystems) and subjected to capillary electrophoresis with the ABI3730 DNA Analyzer (Applied Biosystems). Sanger electropherograms were analyzed by Variant Reporter v1.0 (Applied Biosystems), and the ABI files (.ab1) were visualized with the Chromas software and printed to PDF files to generate vector-based graphics that are presented in the corresponding Figures.

### In silico prediction of RNA splicing

In silico analysis of variants was performed using the splicing prediction module of Alamut Visual (v.2.11.0, Interactive Biosoftware) running four independent algorithms for splice signal detection: SpliceSiteFinder-like (SSF), MaxEntScan (MaxEnt), NNSPLICE and GeneSplicer. The consequences of variants on RNA target motifs bound by splicing proteins were analyzed by the online tool SpliceAid^30^. The deep learning-based tool to identify splice variants SpliceAI^31^ was also used.

### Generation and characterization of patient-specific *KCNQ1* minigene constructs

Minigene constructs were designed to include an insert of 2078 bp corresponding to a *KCNQ1* gene fragment covering the complete sequence between exon 13 and exon 15, which contains the candidate variant c.1686−9 T > C located in intron 13. The insert, which consists of three exons (E13, E14, and E15) and two introns (i13, i14), was generated by high-fidelity amplification (Phusion Hot Start II high-fidelity DNA polymerase, Thermo Scientific) of the index patient’s genomic DNA using the forward primer NotI-545 (GTC TGC GGC CGC GCA AGC GCG GAA GCC TTA CGA TG) and the reverse primer EcoRV-546 (CAG CGA TAT CTT GTC TTC TAC TCG GTT CAG GCG GG). The PCR product was subjected to a double NotI/EcoRV restriction digestion, purified by agarose electrophoresis and eluted from gel slices. The purified insert was directionally cloned in-frame into the NotI and EcoRV sites of the p3XFLAG-Myc-CMV-26 vector (Sigma–Aldrich) for dual-tagged expression of N-terminal 3xFLAG and C-terminal Myc, using standard molecular cloning techniques. This strategy for *KCNQ1* minigene construction preserves the proximal patient-specific endogenous genomic sequence surrounding the variant of interest, and the minigene expression could be analyzed at the mRNA and protein level, after cellular transfection. Before cloning, the presence of the c.1686−9 T > C variant in the purified insert was verified by Sanger sequencing. Plasmid DNA was isolated from transformed and ampicillin-resistant XL1-Blue Supercompetent *E. coli* cells (Stratagene) using the QIAprep Spin Miniprep Kit (Qiagen) and separated by agarose gel electrophoresis to identify positive constructs containing the insert. All positive *KCNQ1* minigene constructs were subjected to full-length insert Sanger DNA sequencing, using the following sequencing primers: 480 (forward from vector, GCA GAG CTC GTT TAG TGA ACC GTC), 481 (reverse from vector, GCA ACT TCC AGG GCC AGG AG), 549 (forward from insert in i13, GCT CAG GTA GCA ACA CCA GCT TCA C), 550 (forward from insert in i13, GAG TGA CCT GGC AGG CCT TAG G) and 551 (forward from insert in i14, CAA ACA GCA GCC ACG GCT CAT A). Plasmids with the reference T or alternative C nucleotide, corresponding to the variant c.1686−9 T > C, were classified as REF or MUT, respectively, and used for expression studies.

### In vitro assay of pre-mRNA splicing by minigene expression

The consequences of the *KCNQ1* variant c.1686−9 T > C on RNA splicing were evaluated by RT-PCR and western blot analysis of HeLa cells transiently transfected with the REF or MUT minigene plasmids, as previously described^32^. The HeLa cell line was purchased from the European Collection of Authenticated Cell Cultures (Sigma–Aldrich) and cultured in the cell culture medium recommended by the manufacturer. Cells were trypsinized at 80% confluence, and cell numbers were determined using an automated cell counter (Countess, Invitrogen) and plated in 12-well culture plates, allowed to attach overnight and transiently transfected with 1.0 µg of plasmid DNA. Cellular transfections with REF or MUT plasmids were performed in triplicated wells using Lipofectamine 3000 (Invitrogen) following the manufacturer’s instructions. The cells were harvested 48 h after transfection. Each cell culture well was processed independently for RNA or protein extraction, and transfection experiments were repeated twice in each assay. All gels and blots in Figures contained a set of samples processed and analyzed in parallel.

### RT-PCR analysis of transfected cells

Total RNA from transfected cells was purified with the RNeasy-Mini Kit (Qiagen) according to the manufacturer’s protocol and evaluated by an automated electrophoresis system (Agilent 2200 TapeStation). All samples showed high quality RNA integrity numbers (RIN > 9). One µg of total RNA was reverse transcribed using SuperScript IV (Invitrogen) and oligo-dT primers following the manufacturer’s recommendations and then subjected to semiquantitative RT-PCR with Taq DNA Polymerase (Invitrogen) and primers located in different exons. Amplification of a 263-bp *KCNQ1* cDNA product was performed with a forward primer located in E13 (547: CAA GCG CGG AAG CCT TAC GAT GT) and a reverse primer in the vector-derived Myc-tag coding sequence (530: CCT CAC AGA TCC TCT TCT GAG ATG AGT). The amplification of endogenous *RPL19* (480 bp) was performed with primers 36 (AAC TCC CGT CAG CAG ATC CG) and 65 (CTT GGT CTC TTC CTC CTT GGA) and used as a cDNA normalization control. Negative controls, including nontransfected cells (NTR), nonreversed transcribed RNA (RT-), and nontemplate were used. RT-PCR products were separated by electrophoresis in 2% agarose gels, visualized by ethidium bromide staining, photographed using VersaDoc 1000 (Bio-Rad), sliced from gel, purified and sequenced with the same primers used in the PCR. The relative intensity of the RT-PCR bands was estimated by gel densitometry (Quantity One, Bio-Rad). Statistical significance for comparisons between RT-PCR bands of the same size of REF and MUT groups (averaged from triplicate transfections, repeated twice) was determined using a two-tailed Student’s *t*-test. A *p* value <0.05 was considered statistically significant.

### Western blot analysis of transfected cells

Cell samples from each well plate were independently homogenized and solubilized in 100 µl of Tris-Glycine SDS sample buffer (Novex Life Technologies) supplemented with complete protease inhibitor cocktail (Roche). Extracted proteins were subjected to SDS-PAGE (Mini-Protean-III, Bio-Rad) using 15% polyacrylamide gels, blotted onto PVDF membranes (Hybond-P, GE Healthcare) and probed with mouse monoclonal anti-FLAG M2 (F3165, Sigma–Aldrich, 1:15000) or anti-Myc-tag (SAB2702192, Sigma–Aldrich, 1:1000) antibodies, followed by incubation with anti-mouse IgG peroxidase antibody (A9917, Sigma–Aldrich, 1:33000). Immunological signals were detected by Super-Signal West Pico PLUS chemiluminescent substrate (Thermo Scientific). Protein extracts from nontransfected cells were used as negative controls. The SeeBlue Plus2 prestained protein standard (Invitrogen) was included in each membrane. Equivalence of protein loading was confirmed by Amido Black 10B staining of blots after immunodetection. Western blot films (Amersham Hyperfilm ECL, GE Healthcare) were scanned with the GS-800 Calibrated Densitometer and analyzed with the image analysis program Quantity One (Bio-Rad). Densitometric analysis was performed as described in the previous Methods subsection.

### RT-PCR analysis of *KCNQ1* endogenous expression

In order to avoid an additional blood extraction from the infant proband, we instead decided to perform endogenous *KCNQ1* expression analysis on blood samples obtained from the proband’s parents, once both of them were genotyped for the candidate *KCNQ1* intronic variant c.1689−9 T > C. Whole blood (2.5 ml) was collected from the index patient’s father and mother into PAXgene Blood RNA Tubes (PreAnalytix) and stored at −80 °C until analysis. Total intracellular RNA was isolated and purified from stabilized whole blood using the PAXgene Blood RNA Kit (PreAnalytix) following the manufacturer’s protocol, which includes an on-column DNase digestion. Quality and quantity of total RNA was evaluated by an automated electrophoresis system (Agilent TapeStation). In both samples, the ratio of the 28 S/18 S ribosomal RNA bands was about 1.0, with an estimated RIN of about 5.7, as calculated by the TapeStation algorithm, representing a medium-quality RNA with initial signs of degradation. First-strand cDNA synthesis was performed using SuperScript IV reverse transcriptase (Invitrogen), 500 ng of total RNA and oligo(dT) primer, following the manufacturer’s protocol. PCR amplification of *KCNQ1* cDNA was performed with Taq DNA Polymerase (Invitrogen) and primers located in different exons, the forward primer in exon 13 (547V2: CAA GCG CGG AAG CCT TAC GAT) and the reverse primer in exon 16 (570V0: TGG TGA AGC ATG TCG GTG ATG AGT), designed to amplify a RT-PCR product of 254 bp. RT-PCR products were processed as described in a previous Methods subsection and each band was purified from gel slices and subjected to Sanger DNA sequencing using the same primers used in the PCR.

### Reporting summary

Further information on research design is available in the Nature Research Reporting Summary linked to this article.

## Supplementary information

Supplementary Information

Reporting Summary

## Data Availability

The authors declare that the main data supporting the findings of this study are available within the article and its Supplementary information. The *KCNQ1* c.1686−9 T > C variant was submitted to the ClinVar database with the accession code SCV001439266. Most materials and reagents used in this study are commercially available. Additional data and/or materials are available upon reasonable request, with some restrictions to protect research participants’ privacy.

## Code availability

No custom codes were generated in this work. Proprietary bioinformatic pipeline (NDNA-Pipeline, v.01) and database (HiC-Mutaciones v.10.0) were used for NGS data collection, analysis, annotation, and filtering of genetic variants. Alamut Visual (v.2.11.0) is commercially available from Interactive Biosoftware. The following publicly available software was used for *in silico* prediction of RNA splicing: SpliceAI (https://github.com/Illumina/SpliceAI) and SpliceAid (http://www.introni.it/splicing.html).
