# Supplementary material for: A cryptic splice-altering KCNQ1 variant in trans with R259L leading to Jervell and Lange-Nielsen syndrome
Source: NPJ Genom Med. 2021 Mar 4;6:21. doi: 10.1038/s41525-021-00183-y (PMC7933243; doi:10.1038/s41525-021-00183-y)
Supplement: Supplementary file 1 — Supplementary Information [file 41525_2021_183_MOESM1_ESM.pdf]

Alamut Visual Splicing Predictions

Gene: **KCNQ1** - Transcript: **NM_000218.2** - Variant: **c.1686-9T>C**
Analysis range: **c.1686-108 (Intron 13) - c.1732+45 (Intron 14) [199 bps]**

Donor Sites

	**SSF [0-100]**	**MaxEnt [0-12]**	**NNSPLICE [0-1]**	**GeneSplicer [0-24]**
Threshold	*≥ 70*	*≥ 0*	*≥ 0.4*	*≥ 0*
Intron 13 – c.1686-90	= 78.04			
Intron 13 – c.1686-33	= 80.14	= 5.29	= 0.65	**4.68 ⇒ 4.83** ***(+3.2%)***
**Exon 14 – c.1732** ℕ	= 85.56	= 8.56	= 0.82	**5.05 ⇒ 5.19** ***(+2.9%)***

ℕatural Splice Site

Acceptor Sites

	**SSF [0-100]**	**MaxEnt [0-16]**	**NNSPLICE [0-1]**	**GeneSplicer [0-21]**
Threshold	*≥ 70*	*≥ 0*	*≥ 0.4*	*≥ 0*
Intron 13 – c.1686-64	= 83.71	= 8.60	= 0.75	**7.23 ⇒ 7.51** ***(+3.9%)***
Intron 13 – c.1686-44	= 71.57	= 1.85		
**Exon 14 – c.1686** ℕ	**91.09 ⇒ 88.38** ***(-3.0%)***	**10.82 ⇒ 10.29** ***(-4.9%)***	**0.99 ⇒ 0.97** ***(-1.7%)***	**7.31 ⇒ 7.13** ***(-2.4%)***
Intron 14 – c.1732+1	= 84.47	= 9.16	= 0.90	**7.37 ⇒ 7.42** ***(+0.7%)***
Intron 14 – c.1732+18	= 78.95	= 4.84		
Intron 14 – c.1732+33	= 72.52			

ℕatural Splice Site

© Interactive Biosoftware - Created by Alamut Visual v.2.11.0 on 15/06/2020

Supplementary Figure 1. Splicing prediction generated by the Alamut software for the *KCNQ1* c.1686-9T>C variant

Nucleotide position

#CHROM 11
POS ID 2798207
REF **T**
ALT **C**, **A** or **G**

SpliceAI results (under default parameters)

ALLELE	SYMBOL	DS_AG	**DS_AL**	DS_DG	DS_DL	DP_AG	DP_AL	DP_DG	DP_DL
SpliceAI=**C**	KCNQ1	0.00	**0.02**	0.00	0.00	-48	9	-24	50
SpliceAI=**A**	KCNQ1	0.01	**0.09**	0.00	0.00	-48	9	-24	50
SpliceAI=**G**	KCNQ1	0.01	**0.16**	0.00	0.00	-48	9	-24	50

Details of SpliceAI INFO field

(as provided in https://github.com/Illumina/SpliceAI)

ALLELE	Alternate allele
SYMBOL	Gene symbol
DS_AG	Delta score (acceptor gain)
**DS_AL**	**Delta score (acceptor loss)**
DS_DG	Delta score (donor gain)
DS_DL	Delta score (donor loss)
DP_AG	Delta position (acceptor gain)
DP_AL	Delta position (acceptor loss)
DP_DG	Delta position (donor gain)
DP_DL	Delta position (donor loss)

Delta score of a variant, defined as the maximum of (DS_AG, DS_AL, DS_DG, DS_DL), ranges from 0 to 1 and can be interpreted as the probability of the variant being splice-altering. In the paper, a detailed characterization is provided for 0.2 (high recall), 0.5 (recommended), and 0.8 (high precision) cutoffs. Delta position conveys information about the location where splicing changes relative to the variant position (positive values are downstream of the variant, negative values are upstream).

Supplementary Figure 2. SpliceAI prediction results. The effects of the T>C variation at *KCNQ1* c.1686-9 (g.2798207) were evaluated. Additionally, the other possible nucleotide changes, T>A and T>G, at that position are also shown. The software returned a near null score (0.02) for the DS_AL (Delta score, acceptor loss) at the canonical acceptor site at position g.2798207+9. Although the other variations (T>A and T>G) are less welcomed than T>C in the polypyrimidine tract, displaying increased DS_AL scores (0.09 and 0.16, respectively), they did not reach the minimum significance level.

a

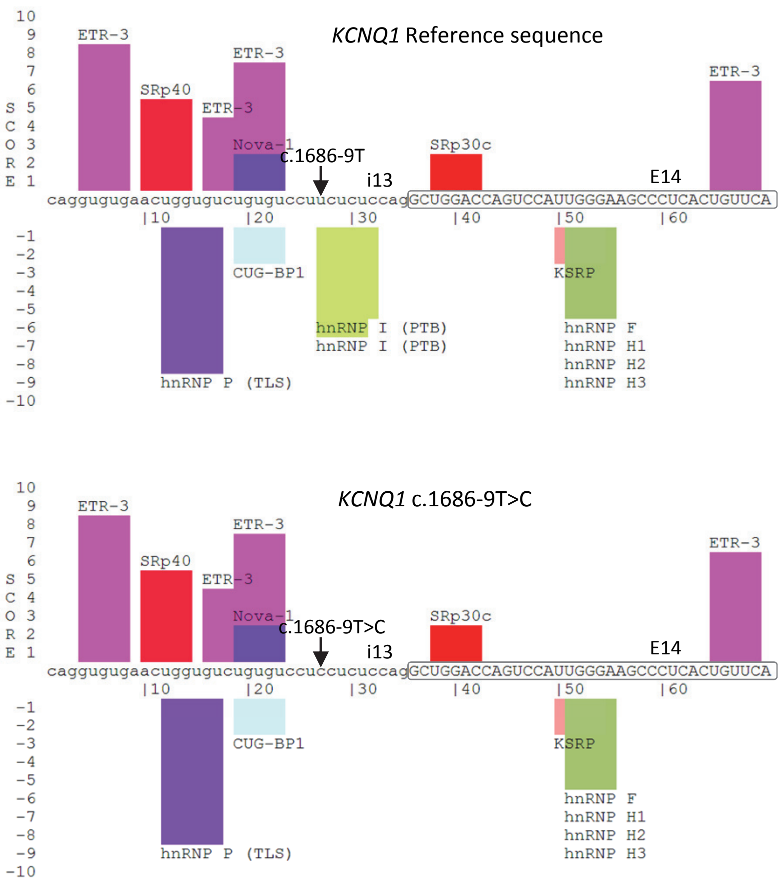

b

Binding sites present ONLY in the first (WILD-TYPE) sequence:

Position	Protein Name	Recognized Sequence	Score	Protein Notes	PubMed ID	Reference	Article notes	Gene/Construct (Target RNA)	Splicing Assay	Binding Assay
27-31	hnRNP I (PTB)	UCUCU	-6	Gene Name and Synonymous: PTBP1, polypyrimidine tract binding protein 1, PTB, PTB2, PTB3, PTB4, pPTB, PTB-1, PTB-T, MGC8461, MGC10830, HNRPI, HNRNPI, HNRNP-I. In the context of CALCA gene, PTB enhances exon 4 inclusion (PMID:9858533). nPTB functionally compensates for PTB and is upregulated when PTB is removed (PMID:17679092).	16179478	Oberstrass FC, Auweter SD, Erat M, Hargous Y, Henning A, Wenter P, Reymond L, Amir-Ahmady B, Pitsch S, Black DL, Allain FH. (2005) Structure of PTB bound to RNA: specific binding and implications for splicing regulation. Science. 309(5743):2054-2057.		Synthesized sequences		NMR, EMSA with recombinant protein
27-32	hnRNP I (PTB)	UCUCUC	-5	Gene Name and Synonymous: PTBP1, polypyrimidine tract binding protein 1, PTB, PTB2, PTB3, PTB4, pPTB, PTB-1, PTB-T, MGC8461, MGC10830, HNRPI, HNRNPI, HNRNP-I. In the context of CALCA gene, PTB enhances exon 4 inclusion (PMID:9858533). nPTB functionally compensates for PTB and is upregulated when PTB is removed (PMID:17679092).	11931771	Charlet-B N, Logan P, Singh G, Cooper TA. (2002) Dynamic antagonism between ETR-3 and PTB regulates cell type-specific alternative splicing. Mol Cell. 9(3):649-658.		Construct of cardiac troponin T TNNT2 [7139] EX4 - INT4 - EX5 - INT5 - EX6.	In vitro splicing with HeLa nuclear extracts. In vivo splicing in HeLa.	UV crosslink, western blot, immunoprecipitation in HeLa nuclear extracts.

No bindig sites are present only in the second (MUTATED) sequence.

Supplementary Figure 3. **SpliceAid analysis of RNA target motifs bound by splicing proteins in the reference or c.1686-9T>C *KCNQ1* sequences.** The junction between intron 13-exon 14 of the reference and c.1686-9T>C *KCNQ1* sequences were used as an input. **a** Graphic output histograms in which each target RNA sequence is represented by a coloured bar whose height is proportional to its binding affinity (score). The software assigned a positive score to the target sequences that facilitate exon definition and a negative score to the target sequences that facilitate intron definition. **b** Description of the binding sites present only in the wild-type sequence. The SpliceAid software predicted that the *KCNQ1* c.1686-9T>C variant abolishes the binding site motif (UCUCU or UCUCUC, the affected nucleotide is underlined) for PTBP1 (polypyrimidine tract binding protein 1, also known as PTB or hnRNP I), located in the intronic region upstream of exon 14. PTBP1 is a ubiquitous RNA-binding protein that regulates splicing. Although PTBP1 is a well-known splicing repressor and does not directly enhance splicing, it can modulate exon inclusion indirectly by preventing the binding of splicing repressors[1,2] .

1. Xue, Y. et al. Genome-wide analysis of PTB-RNA interactions reveals a strategy used by the general splicing repressor to modulate exon inclusion or skipping. Molecular Cell **36,** 996-1006 (2009).
2. Paradis, C. et al. hnRNP I/PTB can antagonize the splicing repressor activity of SRp30c. RNA **13,** 1287-1300 (2007).

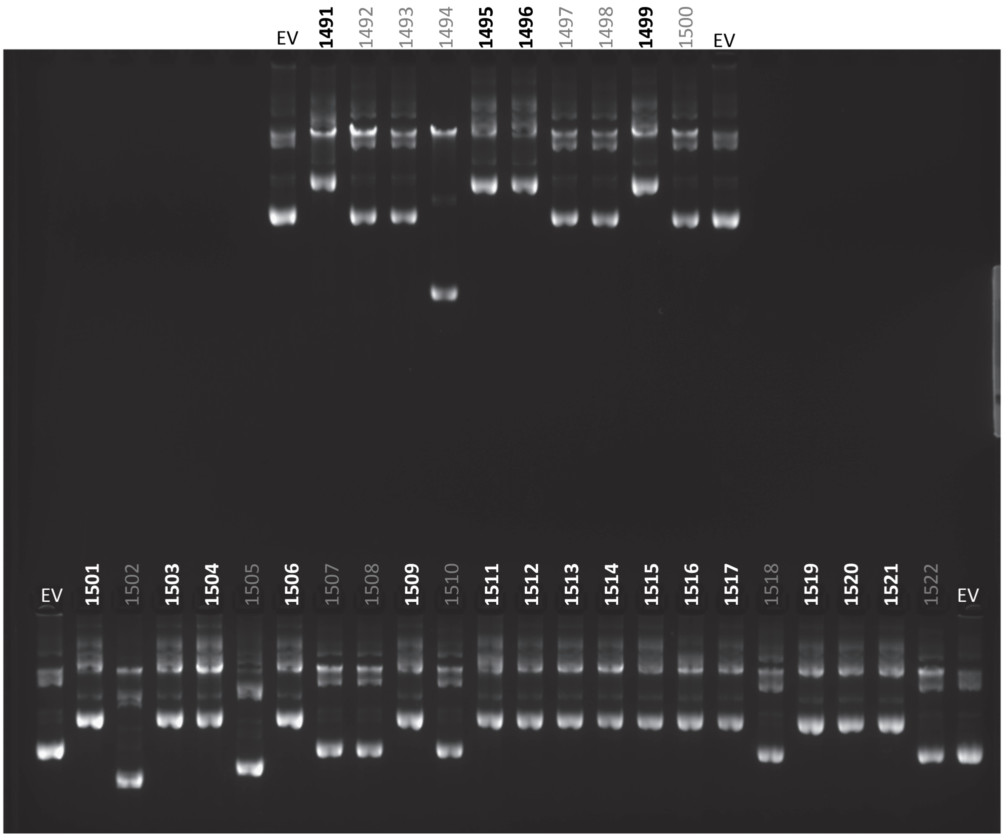

Supplementary Figure 4. **Full size plasmid gel image of Figure 5b**. Identification of positive minigene constructs by agarose electrophoresis of plasmid DNA isolated from 22 ampicillin-resistant bacterial clones (bottom part of the gel). Plasmids with bold numbers, displaying reduced electrophoretic mobility as compared to the empty vector (EV), were identified as positives (containing the insert) and sequenced. Ten additional plasmids (1491-1500) were separated in the upper part of the same gel.

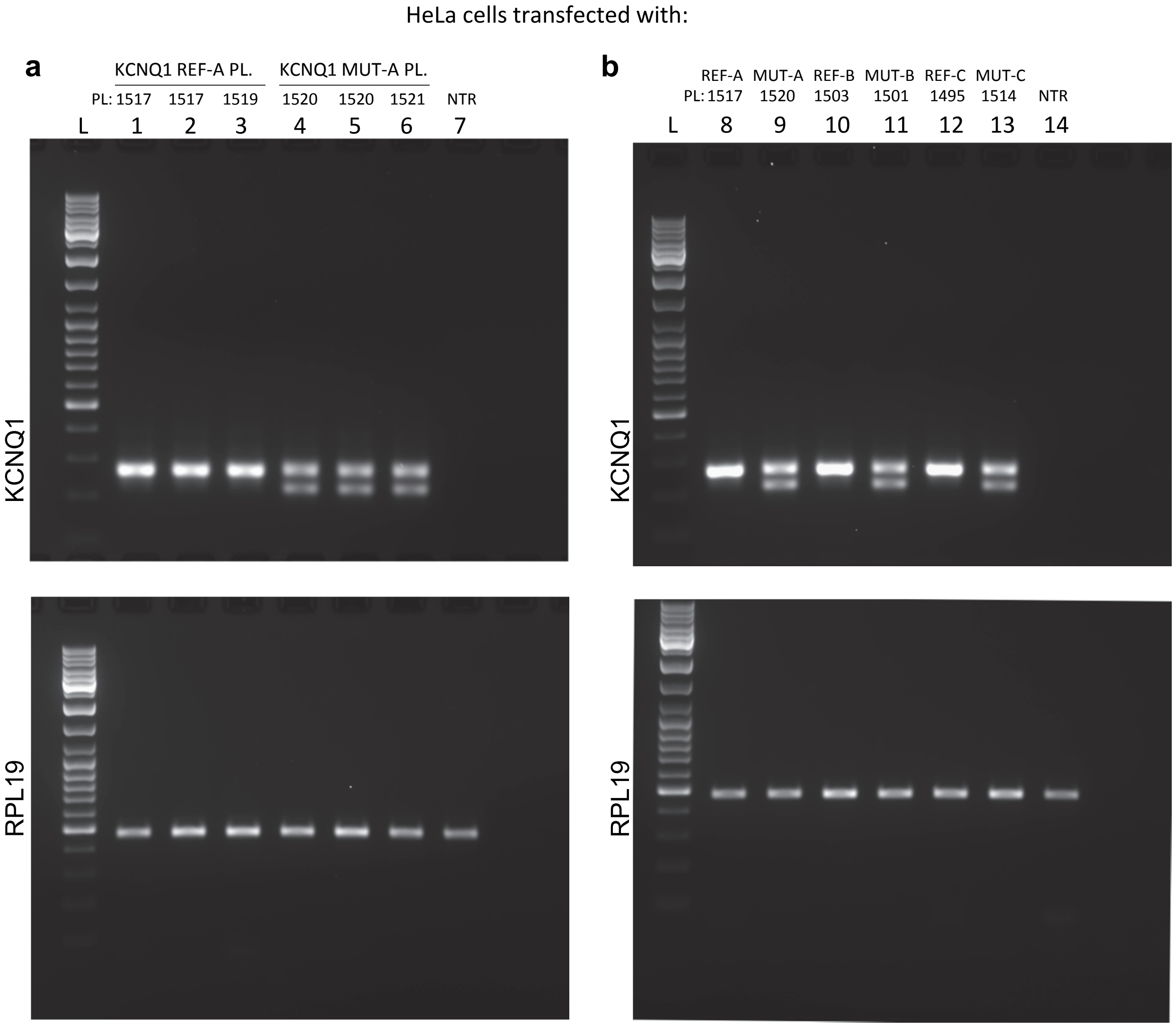

Supplementary Figure 5. Full size RT-PCR gel images of Figure 6. RT-PCR analysis of HeLa cells transfected with *KCNQ1* c.1686-9T>C minigene plasmids. The agarose gel electrophoresis of minigene-derived *KCNQ1* and endogenous *RPL19* RT-PCR products from two transfection experiments are shown in **a** and **b** using the indicated reference (REF) or mutant (MUT) *KCNQ1* minigene plasmids (as described in Fig. 5). Amplification of *KCNQ1* cDNA was performed with a forward primer located in E13 and a reverse primer in the vector-derived Myc-epitope. The 263 bp longer *KCNQ1* bands correspond to the normal mRNA including the exons 13-14-15 and the shorter 216 bp bands correspond to an aberrant mRNA with the complete skipping of exon 14. Each RT-PCR lane (1-14) corresponds to a cell culture well processed independently. The amplification of *RPL19* was used as a cDNA normalization control. NTR non-transfected cells, L DNA ladder.

a RefSeq *KCNQ1* (NM_000218.2)

```
     E12                                     E13
1561 cagtactttgtggccaagaagaaattccagcaagcgcggaagccttacgatgtgcgggac 1620
 521 Q  Y  F  V  A  K  K  K  F  Q  Q  A  R  K  P  Y  D  V  R  D   540

1621 gtcattgagcagtactcgcagggccacctcaacctcatggtgcgcatcaaggagctgcag 1680
 541 V  I  E  Q  Y  S  Q  G  H  L  N  L  M  V  R  I  K  E  L  Q   560
          E14                                              E15
1681 aggaggctggaccagtccattgggaagccctcactgttcatctccgtctcagaaaagagc 1740
 561 R  R  L  D  Q  S  I  G  K  P  S  L  F  I  S  V  S  E  K  S   580
                                                              E16
1741 aaggatcgcggcagcaacacgatcggcgcccgcctgaaccgagtagaagacaaggtgacg 1800
 581 K  D  R  G  S  N  T  I  G  A  R  L  N  R  V  E  D  K  V  T   600

1801 cagctggaccagaggctggcactcatcaccgacatgcttcaccagctgctctccttgcac 1860
 601 Q  L  D  Q  R  L  A  L  I  T  D  M  L  H  Q  L  L  S  L  H   620

1861 ggtggcagcacccccggcagcggcggcccccccagagagggcggggcccacatcacccag 1920
 621 G  G  S  T  P  G  S  G  G  P  P  R  E  G  G  A  H  I  T  Q   640

1921 ccctgcggcagtggcggctccgtcgaccctgagctcttcctgcccagcaacaccctgccc 1980
 641 P  C  G  S  G  G  S  V  D  P  E  L  F  L  P  S  N  T  L  P   660

1981 acctacgagcagctgaccgtgcccaggaggggccccgatgaggggtcctga 2031
 661 T  Y  E  Q  L  T  V  P  R  R  G  P  D  E  G  S  *   676
```

b ΔE14 *KCNQ1*

```
     E12                                     E13
1561 cagtactttgtggccaagaagaaattccagcaagcgcggaagccttacgatgtgcgggac 1620
 521 Q  Y  F  V  A  K  K  K  F  Q  Q  A  R  K  P  Y  D  V  R  D   540

1621 gtcattgagcagtactcgcagggccacctcaacctcatggtgcgcatcaaggagctgcag 1680
 541 V  I  E  Q  Y  S  Q  G  H  L  N  L  M  V  R  I  K  E  L  Q   560
          E15
1681 aggagaaaagagcaaggatcgcggcagcaacacgatcggcgcccgcctgaaccgagtaga 1740
 561 R  R  K  E  Q  G  S  R  Q  Q  H  D  R  R  P  P  E  P  S  R   580
            E16
1741 agacaaggtgacgcagctggaccagaggctggcactcatcaccgacatgcttcaccagct 1800
 581 R  Q  G  D  A  A  G  P  E  A  G  T  H  H  R  H  A  S  P  A   600

1801 gctctccttgcacggtggcagcacccccggcagcggcggcccccccagagagggcggggc 1860
 601 A  L  L  A  R  W  Q  H  P  R  Q  R  R  P  P  Q  R  G  R  G   620

1861 ccacatcacccagccctgcggcagtggcggctccgtcgaccctgagctcttcctgcccag 1920
 621 P  H  H  P  A  L  R  Q  W  R  L  R  R  P  *

1921 caacaccctgcccacctacgagcagctgaccgtgcccaggaggggccccgatgaggggtc 1980
1981 ctga 1984
```

Supplementary Figure 6. Predicted out-of-frame C-terminal KCNQ1 protein encoded by the ΔE14 mRNA. The deduced protein sequence of the reference (**a**) and ΔE14 (**b**) *KCNQ1* transcripts are shown. The skipping of E14 creates a frameshift at the beginning of E15 and a premature termination codon in E16, 79 nucleotides before reaching the natural termination codon. The aberrant protein would lose the last 114 amino acids (underlined) of the C-terminal domain (from Leu563 to Ser676), which would be replaced by 72 new amino acids (double underlined). Thus, the predicted truncated protein is NP_000209.2:p.Leu563Lysfs*73. Each exon (E12-E16) is highlighted with different colors.

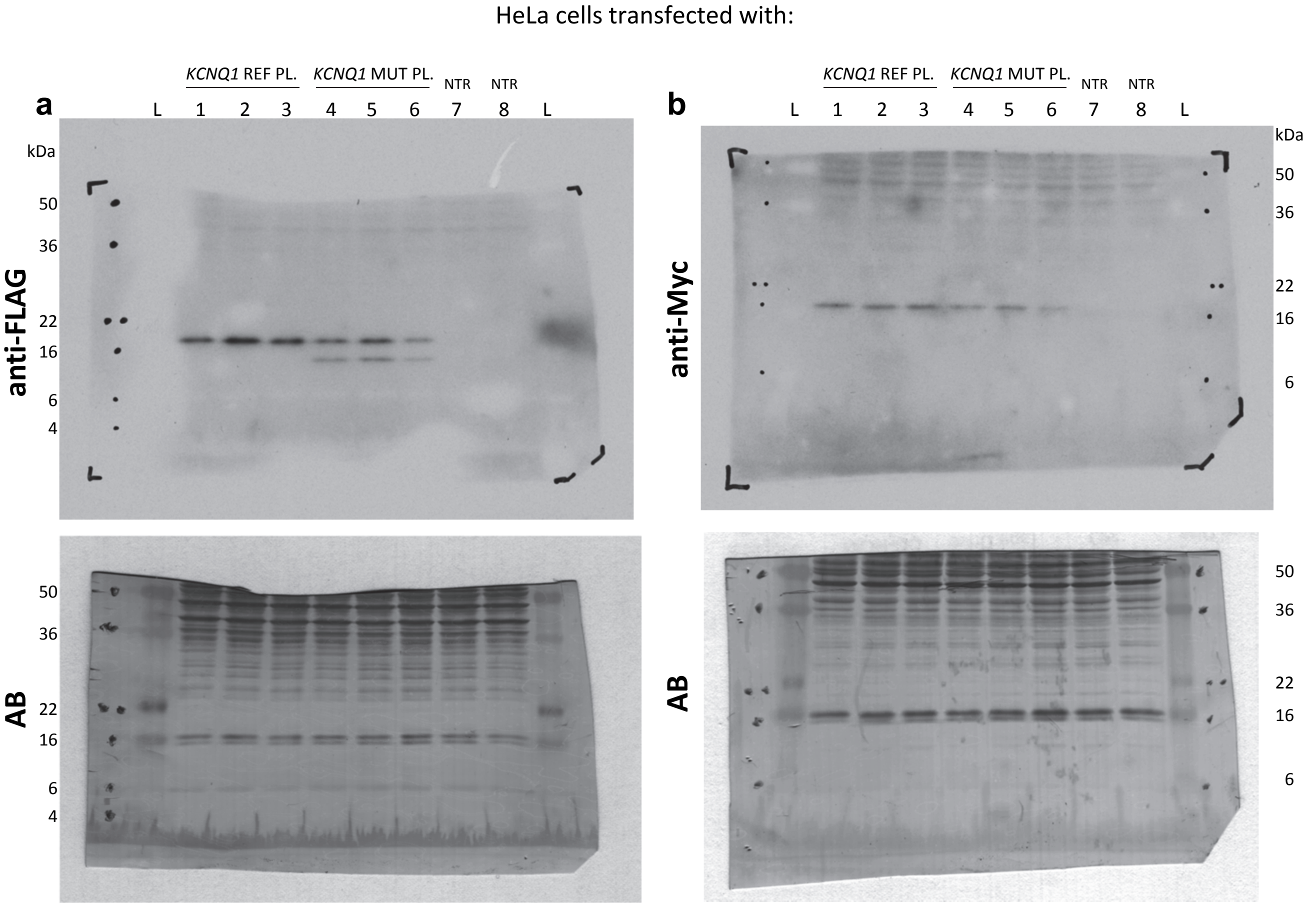

Supplementary Figure 7. Full size Western blot images of Figure 7. Western blot analysis of HeLa cells transfected with *KCNQ1* c.1686-9T>C minigene plasmids. *KCNQ1* Reference (REF) or Mutant (MUT) minigene plasmids (PL) were transfected into HeLa cells (in triplicated wells) as indicated. Cell lysates were analyzed by Western blotting with mouse monoclonal anti-FLAG (**a**) or anti-Myc (**b**) antibodies 48h after transfection. NTR non-transfected cells, AB membrane stained with Amido Black 10B after immunostaining, L protein ladder SeeBlue Plus2 pre-stained protein standard. Molecular weights (kDa) of marker bands are indicated.

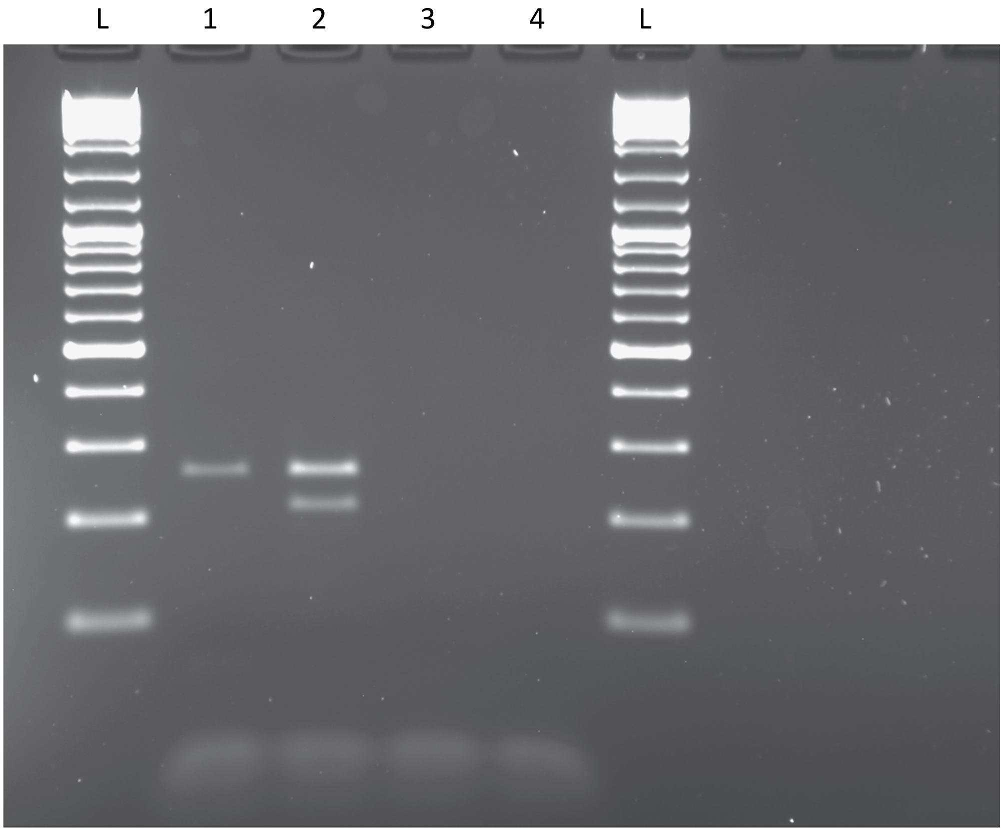

Supplementary Figure 8. Full size RT-PCR image of Figure 8. RT-PCR analysis of the endogenous expression of *KCNQ1* in the blood of the index patient's parents. Agarose gel electrophoresis of RT-PCR products of blood RNA isolated from the index patient's mother (lane 1) and father (lane 2). Amplification of *KCNQ1* cDNA was performed with a forward primer located in E13 and a reverse primer in E16. Negative controls were loaded in lane 3 (-RT) and 4 (non template). The upper bands (254 bp) correspond to the normal *KCNQ1* mRNA and the lower one (207 bp) corresponds to the ΔE14 *KCNQ1* aberrant mRNA**.**
