# Supplementary material for: A cryptic splice-altering KCNQ1 variant in trans with R259L leading to Jervell and Lange-Nielsen syndrome
Source: NPJ Genom Med. 2021 Mar 4;6:21. doi: 10.1038/s41525-021-00183-y (PMC7933243; doi:10.1038/s41525-021-00183-y)
Supplement: Supplementary file 2 — Reporting Summary [file 41525_2021_183_MOESM2_ESM.pdf]

nature research

Corresponding author(s): Mario Torrado

Last updated by author(s): Jan 3, 2021

Reporting Summary

Nature Research wishes to improve the reproducibility of the work that we publish. This form provides structure for consistency and transparency in reporting. For further information on Nature Research policies, see our Editorial Policies and the Editorial Policy Checklist.

Statistics

For all statistical analyses, confirm that the following items are present in the figure legend, table legend, main text, or Methods section.

n/a	Confirmed	
☐	☒	The exact sample size (*n*) for each experimental group/condition, given as a discrete number and unit of measurement
☐	☒	A statement on whether measurements were taken from distinct samples or whether the same sample was measured repeatedly
☐	☒	The statistical test(s) used AND whether they are one- or two-sided<br>*Only common tests should be described solely by name; describe more complex techniques in the Methods section.*
☒	☐	A description of all covariates tested
☒	☐	A description of any assumptions or corrections, such as tests of normality and adjustment for multiple comparisons
☒	☐	A full description of the statistical parameters including central tendency (e.g. means) or other basic estimates (e.g. regression coefficient) AND variation (e.g. standard deviation) or associated estimates of uncertainty (e.g. confidence intervals)
☒	☐	For null hypothesis testing, the test statistic (e.g. *F*, *t*, *r*) with confidence intervals, effect sizes, degrees of freedom and *P* value noted<br>*Give P values as exact values whenever suitable.*
☒	☐	For Bayesian analysis, information on the choice of priors and Markov chain Monte Carlo settings
☒	☐	For hierarchical and complex designs, identification of the appropriate level for tests and full reporting of outcomes
☒	☐	Estimates of effect sizes (e.g. Cohen's *d*, Pearson's *r*), indicating how they were calculated

Our web collection on statistics for biologists contains articles on many of the points above.

Software and code

Policy information about availability of computer code

Data collection	NGS Sequencing data are collected and analyzed using a proprietary bioinformatic pipeline (NDNA-Pipeline, v.01) that includes both sample demultiplexing (using Illumina software) and all the required steps to obtain an annotated variant report, along with the corresponding coverage and quality parameters. The pipeline follows the logical sequence: alignment, alignment refinement and adjustment, variant calling, variant normalization and quality scoring, and generation of coverage statistics by region of interest.
Data analysis	Genetic variants are identified using the reference genome GRCh37/hg19 and reported following the Human Genome Variation Society (HGVS) recommendations (www.hgvs.org). Genetic variants of each study are annotated and filtrated in Health in Code's proprietary database (HiC-Mutaciones v.10.0) according to their potential association with the patient's phenotype. Both analytical sensitivity and specificity of this approach are greater than 99% for single nucleotide variants (SNVs) and insertions/deletions (INDELs) of ≤20 bp. Visualization of NGS data aligned to the human reference genome (GRCh37/hg19) was performed using the Integrative Genomics Viewer (http://software.broadinstitute.org/software/igv/). Health in Code is a clinical genetics company providing UNE-EN-ISO 15189 and CLIA ID 99D2153048 accredited NGS-based services. The following publicly available software was used for in silico prediction of RNA splicing: Alamut Visual v.2.11.0 (Interactive Biosoftware), SpliceAI (https://github.com/Illumina/SpliceAI), SpliceAid (http://www.introni.it/splicing.html).

For manuscripts utilizing custom algorithms or software that are central to the research but not yet described in published literature, software must be made available to editors and reviewers. We strongly encourage code deposition in a community repository (e.g. GitHub). See the Nature Research guidelines for submitting code & software for further information.

Data

Policy information about availability of data

All manuscripts must include a data availability statement. This statement should provide the following information, where applicable:

- Accession codes, unique identifiers, or web links for publicly available datasets
- A list of figures that have associated raw data
- A description of any restrictions on data availability

The authors declare that the main data supporting the findings of this study are available within the article and its Supplementary information. The KCNQ1 c.1686-9T>C variant was submitted to the ClinVar database with the accession code SCV001439266. Most materials and reagents used in this study are commercially available. Additional data and/or materials are available upon reasonable request, with some restrictions to protect research participants' privacy.

Field-specific reporting

Please select the one below that is the best fit for your research. If you are not sure, read the appropriate sections before making your selection.

☒ Life sciences ☐ Behavioural & social sciences ☐ Ecological, evolutionary & environmental sciences

For a reference copy of the document with all sections, see nature.com/documents/nr-reporting-summary-flat.pdf

Life sciences study design

All studies must disclose on these points even when the disclosure is negative.

Sample size	The protocol used for transient transfection of HeLa cells with a number of different constructions in the p3XFLAG-Myc-CMV-26 vector has shown reproducible expression of minigenes. Overall transfection efficiency and equivalent transfection efficiency between biological replicates were previously determined (https://www.hindawi.com/journals/bmri/2018/3536495/). In this study, RNA and protein assays have shown reproducible expression levels of the reference and mutant minigenes using n = 6 independent cell culture wells from two transfection experiments.
Data exclusions	No data were excluded from the analyses.
Replication	Relevant variants identified by NGS were independently confirmed by Sanger DNA sequencing of PCR products. Replication of transfection experiments were successful.
Randomization	Cell culture wells were randomly selected to be transfected with reference or mutant minigenes.
Blinding	Investigators and technical personal involved in NGS and Sanger sequencing were blinded to the identity of the samples. The same investigators were involved in minigene design, construction and expression analysis, thus they were not blinded to group allocation.

Reporting for specific materials, systems and methods

We require information from authors about some types of materials, experimental systems and methods used in many studies. Here, indicate whether each material, system or method listed is relevant to your study. If you are not sure if a list item applies to your research, read the appropriate section before selecting a response.

Materials & experimental systems

n/a	Involved in the study	
☐	☒	Antibodies
☐	☒	Eukaryotic cell lines
☒	☐	Palaeontology and archaeology
☒	☐	Animals and other organisms
☐	☒	Human research participants
☒	☐	Clinical data
☒	☐	Dual use research of concern

Methods

n/a	Involved in the study	
☒	☐	ChIP-seq
☒	☐	Flow cytometry
☒	☐	MRI-based neuroimaging

Antibodies

Antibodies used	Monoclonal ANTI-FLAG M2 antibody produced in mouse, clone M2, purified immunoglobulin, Catalog# F3165, Lot# SLBW9190, Sigma-Aldrich. Monoclonal Anti-Myc tag antibody produced in mouse, clone GT0002, affinity isolated antibody, Catalog# SAB2702192, Lot# 41309, Sigma-Aldrich. Anti-Mouse IgG (Fab specific)–Peroxidase antibody produced in goat, Catalog# A9917, Lot# 098M4827V, Sigma-Aldrich.

Validation	Both epitope tag primary antibodies were validated for Western blot using protein extracts from non-transfected and transfected cells as negative and positive controls, respectively.

Eukaryotic cell lines

Policy information about cell lines

Cell line source(s)	The HeLa cell line (passage +4) was purchased from the European Collection of Authenticated Cell Cultures, Catalogue number 93021013, Lot# 16G007, Sigma Aldrich.
Authentication	The HeLa cell line Lot# 16G007 was authenticated by STR Profiling by the manufacturer (https://www.phe-culturecollections.org.uk). Primary stocks propagated from the original frozen culture were used (passage <+8).
Mycoplasma contamination	The HeLa cell line Lot# 16G007 tested negative for mycoplasma contamination, as documented in the Certificate of Analysis provided by the manufacturer.
Commonly misidentified lines (See ICLAC register)	No commonly misidentified cell line was used.

Human research participants

Policy information about studies involving human research participants

Population characteristics	In this work, we present the genetic characterization of a one-year-old male infant with clinical findings of JNLS, including a prolonged QT interval and chronic bilateral sensorineural deafness. The proband underwent NGS genetic testing to confirm the diagnosis of JLNS and to identify the genetic origin the disease.
Recruitment	As the parents of an infant with JLNS may have long QT syndrome (LQTS), the proband's first-degree relatives were evaluated.
Ethics oversight	Written informed consent was obtained from the minor's parents and all family participants for the genetic analyses and for the publication of anonymized data obtained through the clinical characterization and the scientific research carried out. Ethical approval (nº 1906-20) has been obtained from the Ethics Committee of the Almazov National Medical Research Centre, Saint Petersburg, Russia. Protocol approval is not required. The study was conducted in accordance with the Declaration of Helsinki.

Note that full information on the approval of the study protocol must also be provided in the manuscript.
